# Convergent Evidence That ZNF804A Is a Regulator of Pre-messenger RNA Processing and Gene Expression

**DOI:** 10.1093/schbul/sby183

**Published:** 2018-12-29

**Authors:** Ria M Chapman, Caroline L Tinsley, Matthew J Hill, Marc P Forrest, Katherine E Tansey, Antonio F Pardiñas, Elliott Rees, A Michelle Doyle, Lawrence S Wilkinson, Michael J Owen, Michael C O’Donovan, Derek J Blake

**Affiliations:** 1 Division of Psychological Medicine and Clinical Neurosciences, MRC Centre for Neuropsychiatric Genetics and Genomics, School of Medicine, Cardiff University, Cardiff, UK; 2 College of Biomedical and Life Sciences, Cardiff University, Cardiff, UK; 3 School of Psychology, Cardiff University, Cardiff, UK

**Keywords:** schizophrenia, gene expression, RNA-binding proteins, alternative splicing, autism spectrum disorder, neurodevelopment

## Abstract

Genome-wide association studies have linked common variation in *ZNF804A* with an increased risk of schizophrenia. However, little is known about the biology of ZNF804A and its role in schizophrenia. Here, we investigate the function of ZNF804A using a variety of complementary molecular techniques. We show that ZNF804A is a nuclear protein that interacts with neuronal RNA splicing factors and RNA-binding proteins including RBFOX1, which is also associated with schizophrenia, CELF3/4, components of the ubiquitin-proteasome system and the ZNF804A paralog, GPATCH8. GPATCH8 also interacts with splicing factors and is localized to nuclear speckles indicative of a role in pre-messenger RNA (mRNA) processing. Sequence analysis showed that *GPATCH8* contains ultraconserved, alternatively spliced poison exons that are also regulated by RBFOX proteins. *ZNF804A* knockdown in SH-SY5Y cells resulted in robust changes in gene expression and pre-mRNA splicing converging on pathways associated with nervous system development, synaptic contact, and cell adhesion. We observed enrichment (*P* = 1.66 × 10^–9^) for differentially spliced genes in *ZNF804A*-depleted cells among genes that contain RBFOX-dependent alternatively spliced exons. Differentially spliced genes in *ZNF804A*-depleted cells were also enriched for genes harboring de novo loss of function mutations in autism spectrum disorder (*P* = 6.25 × 10^–7^, enrichment 2.16) and common variant alleles associated with schizophrenia (*P* = .014), bipolar disorder and schizophrenia (*P* = .003), and autism spectrum disorder (*P* = .005). These data suggest that ZNF804A and its paralogs may interact with neuronal-splicing factors and RNA-binding proteins to regulate the expression of a subset of synaptic and neurodevelopmental genes.

## Introduction

Schizophrenia is a highly heritable neuropsychiatric disorder that is often associated with poor quality of life and premature mortality.^1–3^ Risk is conferred by many common alleles of individually weak effect (collectively accounting for up to 40% of disease liability^4^), as well as rare alleles, some of stronger effect. Genome-wide association studies (GWAS) have identified large numbers of common genetic variants associated with an increased risk in schizophrenia,^5–8^ with the most recent study reporting 179 independent associations mapping to 145 independent loci.^7^ One of the first variants implicated in schizophrenia through GWAS was rs1344706, within intron 2 of *ZNF804A*. Following the original GWAS, the association between rs1344706 and schizophrenia has been independently replicated in large meta-analyses of schizophrenia and in a combined analysis of schizophrenia and bipolar disorder.^7–11^ Although GWAS alone do not definitively identify causal variants, rs1344706 is one of only 5 single-nucleotide polymorphisms (SNPs), all intronic, that collectively have a 95% posterior probability of including the causal SNP at this locus.^7^ The risk allele of rs1344706 is associated with decreased messenger RNA (mRNA) levels during the second trimester of fetal brain development.^12^ Allelic variants at rs1344706 have been shown to differentially bind to an unidentified protein in nuclear extracts prepared from SH-SY5Y cells and neural progenitors cells (NPCs)^13^ and are associated with expression of an alternatively spliced *ZNF804A* transcript, lacking the region encoding the zinc finger domain, in fetal brain.^14^*ZNF804A* variants have also been shown to be associated with aspects of working memory performance, cognition, and a range of neuropsychological traits.^15,16^ Furthermore, neuroimaging studies have found that rs1344706 is associated with functional connectivity in healthy individuals and patients with schizophrenia ^17,18^ and neural activity in healthy controls.^19^

At the time of its initial discovery as being associated with schizophrenia, the function of *ZNF804A* was unknown.^20^ Sequence analysis showed that ZNF804A and its 2 paralogs in the human genome, *ZNF804B* and *GPATCH8* (see later), contain a single C2H2-type zinc finger domain that in other proteins has been shown to bind directly to DNA, RNA, protein, and lipids.^21,22^ Several studies have addressed the biological role of ZNF804A. These have implicated ZNF804A in a number of cellular processes including regulation of gene expression, protein translation, neuronal migration, and dendritic spine structure.^23–25^ Transient knockdown of *ZNF804A* in human NPCs was found to result in the differential expression of genes enriched for the Gene Ontology (GO) process *cell adhesion*^26^ whereas stable knockdown of *ZNF804A* in human induced pluripotent stem cells-derived NPCs resulted in the differential expression of genes in the interferon signaling pathway.^27^

Although these studies suggest that ZNF804A may have regulatory activity at the transcriptional or translational level, its precise molecular function is still unknown. Here, we provide the first evidence that ZNF804A interacts with proteins that have defined roles in pre-mRNA processing and alternative splicing, including RBFOX1 that is also associated with schizophrenia.^7^ These data are further corroborated by knockdown of ZNF804A in SH-SY5Y cells, which results in robust changes in gene expression and alternative splicing particularly in synaptic and neurodevelopmental genes, many of which are RBFOX targets. These data suggest that ZNF804A may regulate the expression and splicing of neurodevelopmental genes, some of which have been implicated in the genetic etiology of autism spectrum disorder (ASD) and schizophrenia.

## Materials and Methods

### Yeast 2-Hybrid Screens

Yeast 2-hybrid (Y2H) screens were performed using baits encompassing amino acids 1–620 and 621–1209 of human ZNF804A (NCBI RefSeq NM_194250.1) and amino acids 1–242 and 88–1505 of mouse Gpatch8 (NCBI RefSeq NM_001159492.1) as described previously.^28^ Each bait was screened against an adult mouse brain complementary DNA (cDNA) library (Invitrogen). In addition, the ZNF804A baits were used to screen a human fetal brain (Invitrogen). Gene set enrichment analysis on the list of ZNF804A interactome was performed using the ToppGene suite.^29^ Detailed molecular biology methods can be found in the accompanying supplementary material.

### Cell Culture, RNA Interference, and Microarrays

SH-SY5Y cells were transfected with small interfering RNA (siRNA) duplexes using Lipofectamine RNAi MAX (Life Technologies) according to manufacturer’s instructions. Briefly, SH-SY5Y cells were seeded in 12-well plates (5 × 10^4^ cells per well) in Dulbecco’s modified Eagle’s Medium supplemented with 5% (v/v) fetal calf serum. Adherent cells were transfected with *ZNF804A*-specific siRNA (siZNFA [exon 2], 5ʹ-GGAAAAUACCAUAGCAAAAUU; siZNFB [exon 3], 5ʹ-CCAGGAAAGAUGAAAGAAAUU [Dharmacon]) or glyceraldehyde-3-phosphate dehydrogenase (GAPDH)-specific siRNA (siGAP, 5ʹ-GUCAACGGAUUUGGUGUAUU) at a final concentration of 50 nM (3 wells per condition). The growth media was changed 24 hours post-transfection and the following day the transfection protocol was repeated. After a further 48 hours, cells from the 3 wells were pooled and RNA extracted.

Affymetrix GeneChip Human 1.0 ST microarrays were processed and analyzed at the Central Biotechnology Services facility at Cardiff University. Briefly, total RNA from SH-SY5Y cells was extracted using and labeled with the GeneChip WT Terminal Labeling Kit (Affymetrix) according to the manufacturer’s instructions. Two siRNAs targeting different exons of *ZNF804A* were analyzed alongside 2 negative controls; siGAP- and mock-treated cells (*n* = 4 per condition). Exon array data were analyzed using tools in Partek Genomic Suite 6.4 software (Partek Inc.). Analysis was restricted to the core Meta-probe set that represents 17 881 RefSeq genes and full-length GenBank mRNAs. Probe intensities were quantile normalized and a pre-background adjustment performed based on the guanine-cytosine (GC) content of the probe sequence. Background correction was performed using the robust multiarray analysis algorithm.^30^ To detect any outlier samples, overall gene expression was analyzed by means of a principle component analysis and clustering analysis. All arrays met the quality parameters established by Affymetrix and no outliers were detected. The probe set expression values were summarized to gene-level expression values using the probe set mean. Gene expression changes were identified using a 1-way ANOVA on gene-summarized probe set intensities after multiple test correction using a false discovery rate (FDR) of 0.01.

### Exon Array Identification of Alternative Splicing Events

Alternative splicing events were identified using both probe-level and exon-level intensities. Briefly, pre-analysis filtration a cutoff of log_2_ signal intensity more than 3 was used to remove probe sets not expressed in at least one group.^31^ Alternative splicing events were identified using an alternative splicing 1-way ANOVA on gene-summarized probe set logarithmic intensities. Knockdown type (*ZNF804A* knockdown vs GAPDH knockdown or mock transfection) was chosen as the candidate variable in the ANOVA model to obtain *ZNF804A*-associated splicing events. Alternatively spliced genes (*P* < 1 × 10^–6^, Bonferroni corrected) were selected for downstream analysis.^31,32^ This list was also filtered to exclude genes without Human Genome Organisation (HUGO) symbols and transcript clusters with less than 5 probe sets. We also used probe-level expression changes to discover additional alternatively spliced exons.^33^ Accordingly, we determined further putative alternative splicing events based on the criteria of significantly differentially expressed probe sets (FDR 0.05) in genes that were not differentially expressed (*P*_*differential expression*_ > .05).

### Gene Set Enrichment Analysis

For GO enrichment analysis, lists of differentially expressed (DEX) or differentially spliced genes were entered into the commercial GeneGo MetaCore pathway analysis software (Thomson Reuters) and tested for enrichment in Maps, Diseases, GeneGO process networks, and GO processes using a hypergeometric model. In addition, we also used the publicly available Database for Annotation, Visualization and Integrated Discovery (DAVID) Bioinformatic Resource 6.8 for further pathway analysis.^34^ Enrichment analysis using functional genes sets (RBFOX and CELF4) was performed using Fisher’s exact test implemented in STATA 12.0 or R as described previously.^35^ For all tests, we considered the list of DEX genes (*n* = 546) and differentially spliced genes (*n* = 972) in *ZNF804A*-depleted cells (extended data set 1). Gene sets comprising RBFOX splicing targets, CELF4 and TCF4 and bound genes and transcripts were obtained from Weyn-Vanhentenryck et al and Wagnon et al.^36,37^ Enrichment of DEX and differentially spliced genes at GWAS risk loci was determined using the multi-marker analysis of genomic annotation (MAGMA) package using a window of 35 kb upstream and 10 kb downstream with the whole genome as background.^38,39^ Summary statistics were obtained from the clozapine UK (CLOZUK) and Psychiatric Genomics Consortium’s (PGC) schizophrenia study (CLOZUK + PGC2), the PGC’s bipolar disorder and schizophrenia working group study and the Lundbeck Foundation Initiative for Integrative Psychiatric Research (iPSYCH) common risk variants in ASD study.^7,40,41^ SNPs with information (INFO) scores more than 0.6 were retained as described previously.^7^ Variants within the major histocompatibility complex (MHC) region were removed as in previous studies of enrichment given it is not possible to adequately control for the complex patterns of long range linkage disequilibrium (LD) in this region.^7^ Lists of genes containing de novo variants (loss of function [LoF], missense, and synonymous) identified in patients (schizophrenia, ASD, and intellectual disability [ID]) and controls were obtained from Genovese et al.^42^ Genes that were DEX or differentially spliced in *ZNF804A*-depleted cells were tested for enrichment for de novo variants in schizophrenia, ASD, and ID controls using denovolyzeR.^43^

## Results

### The ZNF804A Interactome

**Fig. 1.**
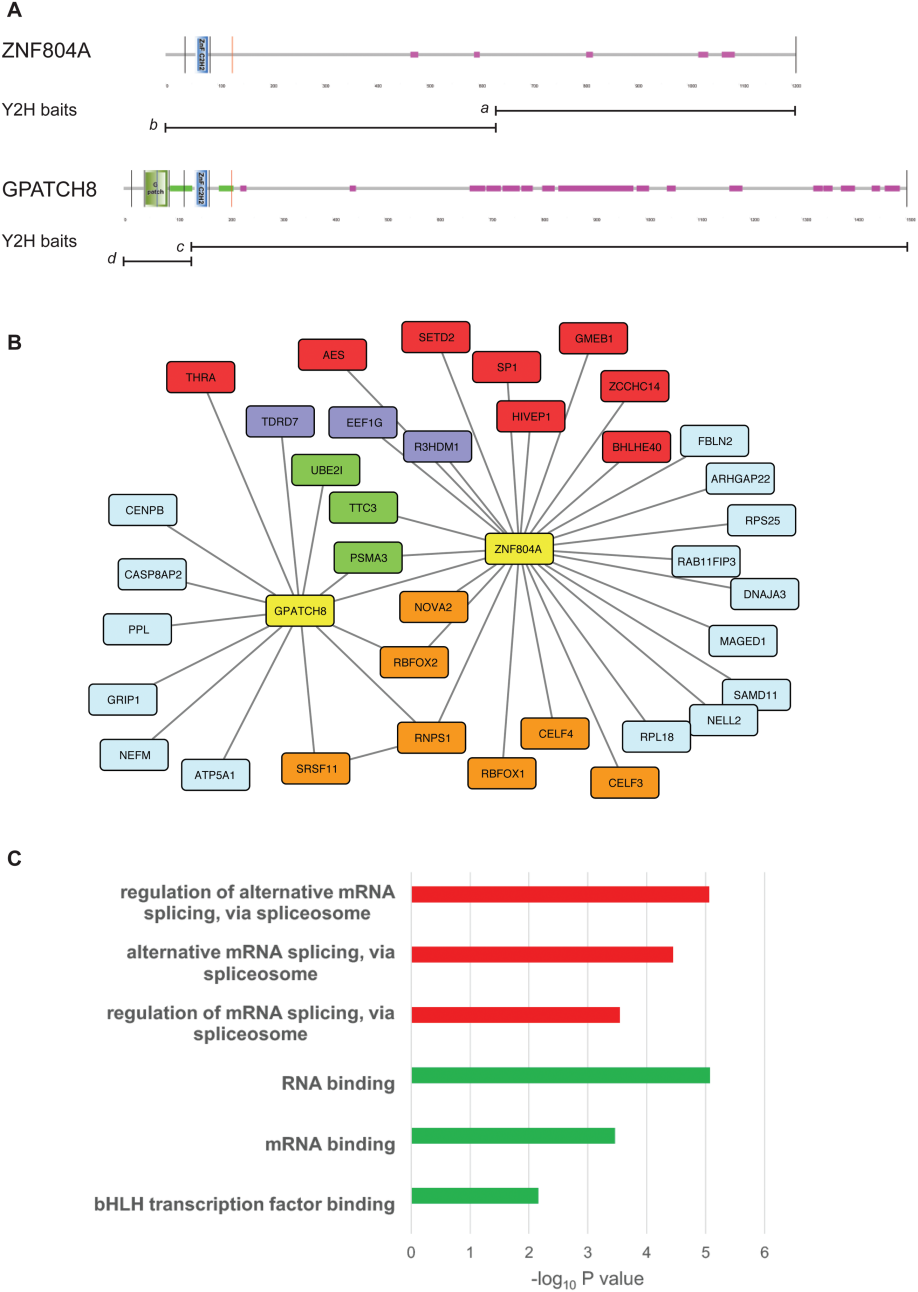
The ZNF804A interactome. Schematic depicting the domain architecture of ZNF804A and its paralog GPATCH8 (A). Images were produced using SMART and are annotated to show protein domains and motifs. GPATCH8 differs from ZNF804A at its N-terminus where the larger protein contains a G-patch domain and an extensive coiled-coil region (green). GPATCH8 also contains a higher proportion of low complexity protein sequence (pink) than its paralog. The bisecting lines show the location of the coding exon boundaries in the 2 proteins. The position of the Y2H baits (*a*-*d*) is shown below each protein. Protein-protein interaction (PPI) network for ZNF804A and GPATCH8 (B). PPI was produced in CYTOSCAPE and shows combined Y2H data from the four baits depicted in panel A. Interacting genes are color-coded according to molecular function; baits, yellow; messenger RNA processing and binding, orange; RNA-associated, purple; transcription, red; ubiquitin-proteasome, green; unspecified, light blue. Gene Ontology (GO) enrichment for the entire ZNF804A interactome (C). Enrichment analysis was performed on the list of interactors using ToppGene. The top 3 statistically significant GO molecular function (red) and GO biological processes (green) are shown after Bonferroni correction.

To gain insights into the function of ZNF804A, we used the Y2H system to find ZNF804A-interacting proteins. Two baits spanning the entire coding sequence of human ZNF804A were used to screen human embryonic brain and adult mouse brain cDNA libraries (figure 1A). These screens identified multiple interacting clones from each library (figure 1B), including several proteins with nucleic acid-binding domains (splicing and transcription factors) and components of the ubiquitin-proteome system (UPS). The splicing factors RNPS1 and RBFOX2 and a subunit of the proteasome (PSMA3) were identified in both the human and mouse libraries. In addition to RNPS1 and RBFOX2, the splicing factors, RBFOX1 and NOVA2, and the RNA-binding proteins CELF3 (BRUNOL1) and CELF4 (BRUNOL4) were found to interact with ZNF804A in yeast. Interestingly, the ZNF804A paralog GPATCH8 was found in the ZNF804A interactome, suggesting a physical interaction between these proteins (figure 1B).

GPATCH8 contains an N-terminal G-patch domain and coiled coil regions flanking the C2H2 zinc finger domain. G-patch domains are found in some RNA-binding proteins including components of the spliceosomal complex.^44^ Having found evidence for a physical interaction between GPATCH8 and ZNF804A, we re-screened the adult mouse brain cDNA library to find additional GPATCH8-interacting proteins. These experiments showed that GPATCH8 interacts with UPS components (PSMA3 and UBE2I) and the splicing factors RNPS1 and SRSF11. The interaction between GPATCH8 (KIAA0553), RNPS1, and SRSF11 (p54) has been reported previously in HeLa cells demonstrating the efficacy and reproducibility of the Y2H system in identifying robust protein-protein interactions.^45^ The entire ZNF804A interactome was used for pathway analysis using the ToppGene software suite (figure 1C).^29^ This analysis showed that the ZNF804A network was enriched for several GO processes including regulation of alternative mRNA splicing, via spliceosome (GO:0000381, *P* = 8.78 × 10^–6^) and RNA binding (GO:0003723, *P* = 8.47 × 10^–6^).

To further examine the molecular function of ZNF804A and its paralog GPATCH8, we expressed constructs encoding each protein in heterologous cells. We were unable to detect the endogenous ZNF804A protein in brain (wild-type and *Zfp804a* mutant mice) or SH-SY5Y neuroblastoma cells (see later) using either commercially available antibodies or antisera developed “in-house.” However, ZNF804A was detected in transfected cells following proteasome inhibition with lactacystin (supplementary figure S1A). These data are consistent with the multiple interactions of ZNF804A and GPATCH8 with UPS components and also suggest that ZNF804A levels may be tightly regulated. Confocal microscopy revealed that ZNF804A was predominantly localized to the nucleus of transfected cells (supplementary figure S1B). For comparative purposes, we also cloned the murine ortholog of GPATCH8 (Gpatch8) from brain. Sequence analysis revealed that *Gpatch8* contained 2 poison cassette exons flanking exon 4 (encoding the G_patch domain), whose inclusion would result in premature translational termination (supplementary figure S1C). Poison exons are ultraconserved sequences containing in-frame stop codons that prematurely terminate translation.^46^ All members of the serine/arginine-rich family of splicing factors such as SRSF11 (figure 1B) and SRSF2 (SC35; supplementary figure S1E) contain poison exons that are thought to autoregulate protein levels by self-splicing, coupling alternative splicing to nonsense-mediated decay of the unspliced transcript.^46^ G_patch domain-containing proteins are enriched for GO processes including nucleic acid binding and mRNA processing (supplementary figure S1D). Finally, Gpatch8 was only detected in the nucleus of transfected cells where it appeared to be localized to nuclear speckles, subnuclear structures (labeled with SC35) that are enriched in pre-mRNA splicing factors (supplementary figure S1E).^47^ Together, our data suggest that ZNF804A and GPATCH8 are involved in pre-mRNA processing and alternative splicing.

### ZNF804A Knockdown in SH-SY5Y Cells

To test the hypothesis that ZNF804A may be involved the regulation of gene expression and pre-mRNA processing, we used siRNAs to knockdown endogenous *ZNF804A* in the SH-SY5Y neuroblastoma cell line. SH-SY5Y cells express similar levels of *ZNF804A* to those found in the brain and have been used for functional analysis of many neuropsychiatric risk genes including ZNF804A itself.^24,35,48^ Two siRNA duplexes, siZNFA (exon 2) and siZNFB (exon 3), were designed to knockdown *ZNF804A*. In addition to the siRNAs targeting *ZNF804A*, we used a duplex targeting *GAPDH* (siGAP) and mock-transfected cells as controls. The siGAP duplex was chosen because it has minimal off-target effects while activating the RNA-induced silencing complex pathway in transfected cells. RNAi-mediated knockdown of each gene was determined by quantitative polymerase chain reaction (qPCR) on first strand cDNA prepared from each experimental condition. We observed an average 81% (SD, 8.4) and 77% (SD, 8.8) reduction in ZNF804A mRNA using siZNFA and siZNFB, respectively when compared with mock-treated and siGAP-treated cells (supplementary figure S2A). Similarly, we observed an 86% (SD, 3.2) reduction in GAPDH mRNA using siGAP (supplementary figure S2B). Thus, transfection of each duplex resulted in robust knockdown of its target.

RNA samples from siRNA-treated cells and controls (siZNFA, siZNFB, siGAP, and mock-transfected; *n* = 4) were converted to cDNA and hybridized to the exon array. Data quality assessment identified no outlier arrays using Expression Console Software, thus data from all arrays were included in the subsequent analyses. Signal intensity data from knockdown of ZNF804A using siZNFA and siZNFB were grouped before analysis to give the siZNFPooled dataset. This ensures that any changes seen were a result of ZNF804A knockdown and not due to off-target effects of the individual siRNAs. Analyzing the 17 881 genes represented in the core probe set list we identified a total of 579 genes (FDR 0.01) that showed differential expression between siZNFPooled and siGAP (extended data set 1). By contrast, only 47 genes were DEX following *GAPDH* knockdown. qPCR was used to validate the gene expression changes in *ZNF804A*-depleted cells that were identified using the exon array. Eight DEX genes (*SPARC*, *FSTL4*, *NPY*, *PDK1*, *EGR1*, *TMEFF2*, *CCL2*, and *EFNB2*) that had a single isoform were selected for independent validation by qPCR. In each case, qPCR levels were in close agreement with the fold change detected on the microarray thereby confirming the utility of the exon array to detect robust gene expression changes in *ZNF804A*-depleted cells (supplementary table S2).

Having shown that ZNF804A interacts with proteins involved in pre-mRNA processing (figure 1), we used the microarray data to search for alternatively spliced exons that were differentially expressed in *ZNF804A*-depleted cells. We investigated the exon-level changes following *ZNF804A* knockdown using 2 methods. First, we applied an alternative splicing ANOVA to gene-summarized probe set intensities along with several filtration criteria. We identified candidate alternative splicing events in 448 genes (extended data set 1). Second, we selected individual probe sets with differential expression in genes that were not DEX.^33,49^ This analysis identified 566 differentially expressed individual probe sets after *ZNF804A* knockdown (extended data set 1). In control experiments, we compared differential splicing in the *GAPDH*-knockdown cells with mock-transfected cells using gene-summarized probe set intensities as described previously. Consistent with the rationale that *GAPDH* encodes a metabolic enzyme that is not known to have a role in alternative splicing, we identified only 13 differentially spliced genes in siGAP-treated cells compared with the mock-transfected controls using the same statistical parameters (data not shown).

**Fig. 2.**
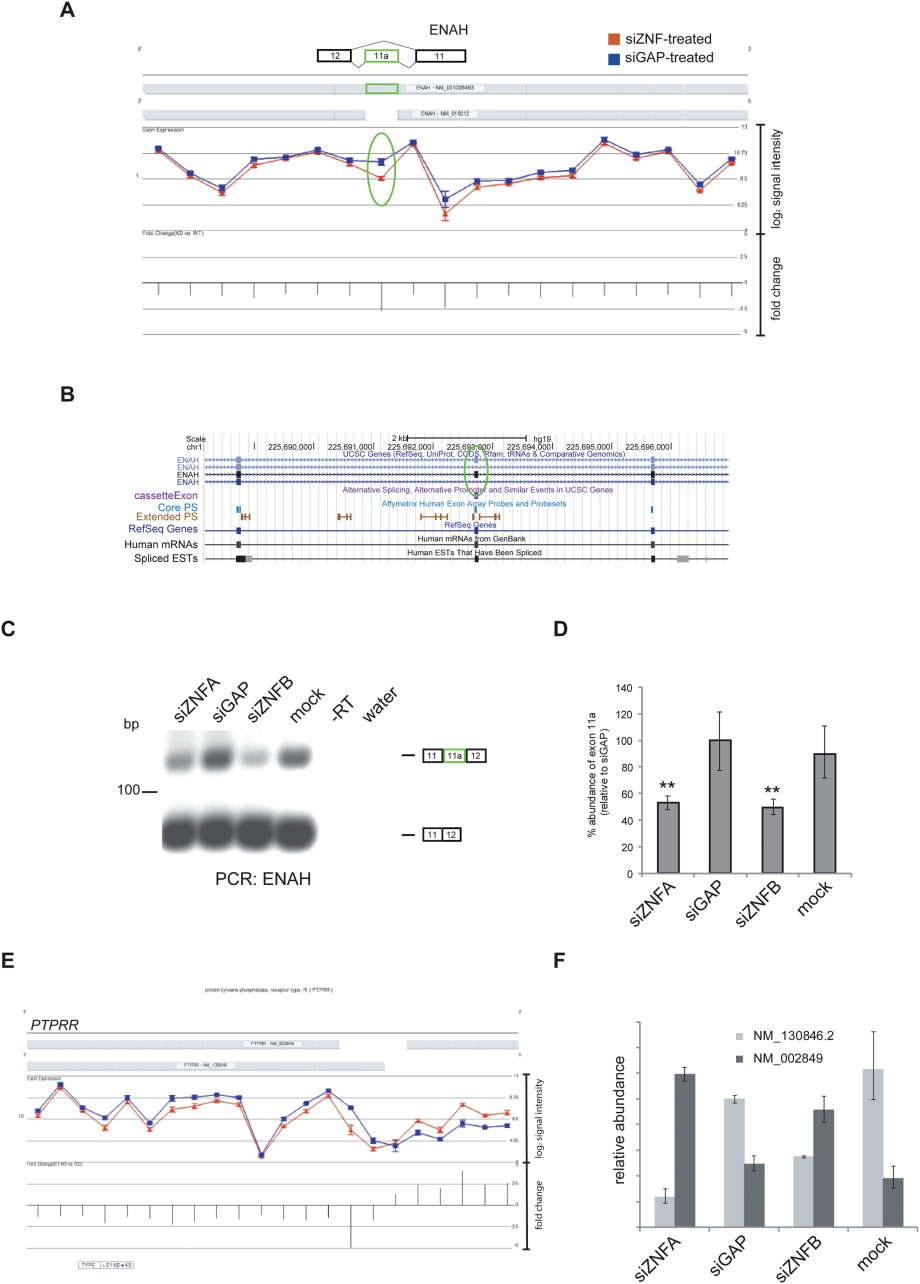
Alternative splicing events in *ZNF804A*-depleted cells. Geneview for *ENAH* after *ZNF804A* knockdown (A). Following *ZNF804A* knockdown there was increased exclusion of the exon 11a (highlighted with an oval). Screenshot showing the genomic region containing *ENAH* exon 11a and flanking exons displayed using the UCSC Genome Browser (B). Reverse transcription polymerase chain reaction (RT-PCR) using primers complementary to the constitutive exons flanking *ENAH* exon 11a (C). Quantitative PCR (qPCR) using primers complementary to exon 11a and a control pair complementary to another region on the transcript (D). The expression of exon 11a was compared between samples using the ∆∆Ct method with the control primer set as the endogenous normalizer. The bar graph shows the percentage abundance of exon 11a relative to the glyceraldehyde-3-phosphate dehydrogenase-specific siRNA (siGAP)-treated sample. The error bars represent the standard deviation of the raw Ct values. The significance was assessed using a 1-way ANOVA and Tukey’s post-hoc test. ** *P* < .05. *PTPRR* isoform switch in *ZNF804A*-depleted cells (D). *PTPRR* geneview showed a switch in transcript variant use in *ZNF804A*-depleted cells. qPCR using the primers complementary to each transcript of *PTPRR* (E). The expression of each *PTPRR* transcript was compared between samples using the ∆∆Ct method with *beta actin* (*ACTB*) set as the endogenous normalizer. The bar graph shows the relative abundance of each *PTPRR* transcript relative to the abundance of the canonical transcript (NM_130846.2) in the siGAP-treated sample. NM_002849 (canonical membrane bound).

**Fig. 3.**
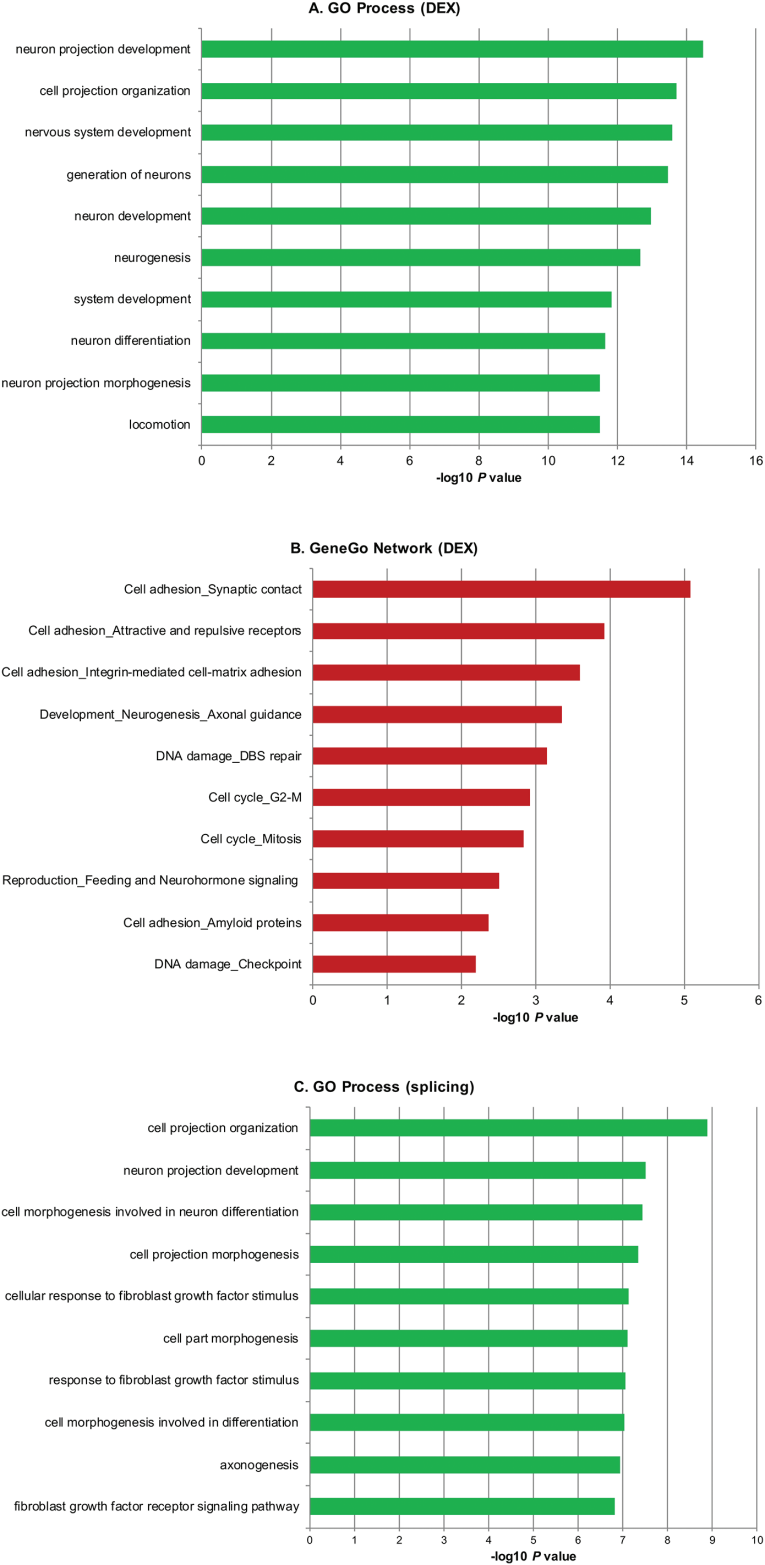
Enrichment analysis of differentially expressed (DEX) and differentially spliced genes in *ZNF804A*-depleted cells. The lists of DEX and differentially spliced genes between *ZNF804A*-specific siRNA-treated and glyceraldehyde-3-phosphate dehydrogenase-specific siRNA-treated samples (FDR 0.01) were imported into GeneGo Metacore. The bar charts show the top ranked Gene Ontology (GO) Processes (A, DEX) and (C, alternative splicing) and GeneGo Process Networks (B, DEX) identified by the enrichment analysis after multiple test correction (FDR 0.05). In all cases, only ontologies that pass multiple test correction are shown.

qPCR and reverse transcription PCR (RT-PCR) were used to validate differential alternative splicing events identified using both of the methods described earlier. We focused on genes that contained previously annotated splicing events by comparing the Partek geneview for each gene with their the University of California, Santa Cruz (UCSC) entry. For example, the geneview of *ENAH* (enabled homologue—actin regulator) showed increased exclusion of exon 11a (chr1:225692693-225692755) following *ZNF804A* knockdown (*P* = 8.2 × 10^–4^; fold change = –2.69; 1-way ANOVA) and that the *ENAH* transcript was not differentially expressed (figure 2A). This alternative splicing event was also annotated in the UCSC RefSeq database indicating that it corresponds to a sequence-validated event in a proportion of *ENAH* transcripts (figure 2B) and is known to be regulated by RBFOX splicing factors.^50,51^ The results of RT-PCR were consistent with those from the microarray analysis, with a reduction in the longer, exon 11a containing *ENAH* transcript in *ZNF804A*-depleted cells (figure 2C). These data were further confirmed by qPCR, which showed approximately 50% reduction in *ENAH* exon 11a inclusion after *ZNF804A* knockdown relative to both controls (*P* < .01; figure 3D). Together, these data validate the exon array, showing that exon 11a of *ENAH* is differentially spliced (excluded) in *ZNF804A*-depleted cells.

In addition to changes in cassette exon splicing, the alternative splicing ANOVA identified changes in alternative transcript usage after *ZNF804A* knockdown. The geneview of *PTPRR* (encoding protein tyrosine phosphatase receptor type R) showed differential 5ʹ-end usage, indicative of a switch in transcript usage between transcripts encoding the soluble and membrane bound isoforms of the receptor in *ZNF804A*-depleted cells (figure 2E).^52^ This switch in transcript variant usage was confirmed by qPCR and showed that the membrane bound and soluble isoforms of PTPRR arise from the differential use of alternative transcriptional start sites as described previously (figure 2F).^53^ We also confirmed several other differential alternative splicing events in *ZNF804A*-depleted cells (supplementary table S3).

### Pathway and Gene Set Analysis for DEX and Differentially Spliced Genes in ZNF804A-Depleted Cells

We used the GeneGo MetaCore bioinformatics package and the DAVID bioinformatics resource to search for pathways and networks enriched for genes with altered expression or splicing in *ZNF804A*-depleted cells. MetaCore was chosen because the analysis software utilizes both GO processes and GeneGo’s manually curated processes and pathways. Of the 579 DEX genes, 547 had known HUGO IDs and were used for enrichment and pathway analysis. MetaCore enrichment analysis of the DEX genes showed that the most significant GO biological processes were neuron projection development (*P* = 3.35 × 10^–15^), cell projection organization (*P* = 1.96 × 10^–14^), and nervous system development (*P* = 2.66 × 10^–14^) (figure 3A). Enrichment analysis using GeneGo networks highlighted synaptic contact as an element of cell adhesion (*P* = 8.39 × 10^–6^) and axonal guidance as an element of neurogenesis and development (*P* = 4.5 × 10^–4^) (figure 3B). Similarly, we used MetaCore to search for GO processes associated with differentially spliced genes in *ZNF804A*-depleted cells. For these analyses, we used both sets of alternatively spliced genes identified using the gene-level and exon-level ANOVAs. This analysis identified GO processes relating to neuronal and cell projection organization and morphogenesis as enriched for differential splicing events in *ZNF804A*-depleted cells (figure 3C). Similar results were obtained using DAVID (extended data set 1).

Having found that RBFOX1/2 and CELF4 directly interact with ZNF804A, we determined whether DEX and differentially spliced genes in *ZNF804A*-depleted cells were enriched for RBFOX and CELF4 targets. We found that genes that were differentially spliced in *ZNF804A*-depleted cells were highly enriched for RBFOX targets (*P* = 3.31 × 10^–9^, OR 2.32) whereas no enrichment was found among the DEX genes (table 1). By contrast, no enrichment was observed for DEX or differentially spliced genes in *ZNF804A*-depleted cells and transcripts bound by CELF4 (table 1).

**Table 1.** Enrichment for Differentially Expressed (DEX) and Differentially Spliced Genes in *ZNF804A*-Depleted Cells Across Selected Functional Gene Sets

Gene set	DEX genes				Differentially spliced genes			
	*P* value	*P* corr.	OR	95% CI	*P* value	*P* corr.	OR	95% CI
RBFOX targets	.48	ns	0.81	0.46–1.35	4.14 × 10^–10^	1.66 × 10^–9^	2.32	1.80–2.96
CELF4 targets	.41	ns	1.09	0.88–1.35	.19	ns	1.11	0.95–1.30

*Note*: gene sets were collated from the original publication; RBFOX targets defined by an integrative modeling approach and transcripts bound by the CELF4 RNA-binding protein ^36^^,^^37^. *P* values and OR were calculated using Fisher’s exact test and corrected (Bonferroni) for multiple testing. CI, confidence interval; ns, not significant.

Given the extensive evidence for pleiotropy across neuropsychiatric disorders,^2^ we examined the enrichment of DEX and differentially spliced genes for common variants associated with schizophrenia, schizophrenia and bipolar disorder, and ASD. MAGMA was used to test for common variant association based on data from the most recent GWAS^7,40,41^ correcting for gene length and LD.^38^ We found enrichment of differentially spliced genes (in *ZNF804A*-depleted cells) among common variant alleles associated with schizophrenia (*P* = .014), bipolar disorder and schizophrenia (*P* = .003), and ASD (*P* = .005). By contrast, no enrichment was found between DEX genes among common variant alleles in the three GWAS described previously. Finally, we used denovolyzeR^43^ to test for enrichment of DEX and differentially spliced genes among de novo mutations in schizophrenia, ASD, and ID.^42,54^ Differentially spliced genes were enriched for all classes of nonsynonymous mutation in ASD, including the most damaging, LoF mutations (*P* = 6.25 × 10^–7^, enrichment 2.16) (table 2). There was also enrichment (*P* = 1.92 × 10^–3^, enrichment 2.31) for genes harboring LoF mutations in ASD and DEX genes in ZNF804A-depleted cells (supplementary table S4). No enrichment was observed between any class of schizophrenia, ID-associated and control/sibling de novo mutations and differentially spliced or DEX genes in *ZNF804A*-depleted cells (table 2; supplementary table S4).

## Discussion

Despite strong support for the candidacy of *ZNF804A* as a schizophrenia susceptibility gene, very little is known about the function of the protein and how alteration in its function or expression might contribute to the molecular pathology of the disorder.^55^ To address this, we examined the function of ZNF804A at the molecular level, using a combination of techniques to identify ZNF804A-interacting proteins and ZNF804A-regulated genes in a cellular model. We show that ZNF804A and its paralog GPATCH8 interact with a number of RNA-binding proteins and pre-mRNA splicing factors, including members of the RBFOX family that regulate alternative mRNA splicing in neurons.^56^ Knockdown of *ZNF804A* resulted in differential expression of genes involved in nervous system development and synaptic contact and was associated with changes in alternative splicing of genes that are also RBFOX targets. Collectively, these data suggest that ZNF804A associates with a range of RNA-binding proteins to regulate pre-mRNA processing in neuronal cells.

In this study, we show for the first time that ZNF804A interacts with proteins involved in pre-mRNA processing, transcriptional regulators, and UPS components (figure 1B). Notably, ZNF804A was found to interact with splicing factors including RBFOX1/2 and mRNA-binding proteins such as CELF3/4.^56^ RBFOX1 has recently been implicated in schizophrenia in the largest published GWAS of the disorder^7^ and moreover, the targets of both RBFOX proteins and CELF4 are enriched for rare disruptive exonic mutations in people with schizophrenia.^42^ Given the number of RNA-binding proteins that interact with ZNF804A, we propose that ZNF804A is involved mRNA metabolism and alternative splicing. The interaction between ZNF804A and its paralog GPATCH8 lends further support to this hypothesis as follows. The G_patch is an RNA-binding domain that is required for efficient pre-mRNA splicing and splice site selection.^44,57,58^ We found that GPATCH8 in common with other splicing factors contains ultraconserved poison exons that, in other genes, form part of an autoregulatory network coupling alternative splicing to nonsense-mediated decay.^46^ RBFOX sites flank both alternatively spliced poison exons in *GPATCH8* suggesting that RBFOX proteins also regulate alternative splicing of *GPATCH8*.^37^ Collectively, our data suggest that ZNF804A and its paralogs are likely to be involved in the regulation of gene expression at the level of pre-mRNA splicing but do not preclude a role for ZNF804A in translational regulation as recently reported by Zhou et al.^25^

To further investigate the role of ZNF804A in gene regulation (expression and splicing), we used exon arrays to determine the effect of acute *ZNF804A* depletion on the transcriptome of SH-SY5Y neuroblastoma cells. GO enrichment analysis showed that DEX genes clustered into several categories related to neurodevelopment, cell adhesion, and more specifically synaptic adhesion complexes (figure 3A and B). Our results are broadly consistent with those of Hill et al^26^ who showed that knockdown of *ZNF804A* in NPCs resulted in differential expression of genes enriched for the GO process “cell adhesion.”. Furthermore, knockdown of *ZNF804A* in neurons has recently been shown to attenuate neurite outgrowth and promote loss of dendritic spines potentially mediated by reduction in the levels of the cell adhesion protein neuroligin-4.^24^

Alternative splicing generates proteomic diversity and has previously been proposed to be an important contributory process to the molecular pathogenesis of several neuropsychiatric disorders.^59^ Following the rationale that direct interaction between ZNF804A and a range of splicing factors may modulate alternative splicing patterns of some genes, we used the exon arrays to look for changes in alternative splicing in *ZNF804A*-depleted cells. We found that *ZNF804A* knockdown is also associated with robust changes in alternative splicing (figure 3, extended data set 1). Collectively, differentially spliced genes in *ZNF804A*-depleted cells are also enriched for RBFOX targets, again suggesting that both proteins may interact to regulate the splicing of a subset of neuronal genes (table 1).

Finally, we used gene set enrichment analysis to search for associations between DEX and differentially spliced genes in *ZNF804A*-depleted cells and genes implicated in several common neurodevelopmental disorders. We found that genes harboring de novo mutations (missense and LoF) in ASD show enrichment for differentially spliced (and to a lesser extent DEX among LoF variants) genes in *ZNF804A*-depleted cells (table 2). Common and rare variants implicated in schizophrenia, ASD, and ID are also enriched for RBFOX targets.^7,54,60–63^ Furthermore, *RBFOX1* copy number variants have been reported in ASD^64^ whereas *RBFOX1* common variants have recently been shown to confer increased risk of schizophrenia.^7^ The gene set of differentially spliced genes in *ZNF804A*-depleted cells was also enriched for common variant alleles associated with schizophrenia, bipolar disorder and schizophrenia, and ASD. These data suggest that *ZNF804A* may contribute to neurodevelopmental disease risk through splicing regulation.

**Table 2.** Gene Set Enrichment for Differentially Spliced Genes in *ZNF804A*-Depleted Cells and De Novo Disease Variants.

Gene set	Observed	Expected	Enrichment	*P* value	*P* corr.
SCZ_syn	23	22	1.05	.444	ns
SCZ_mis	53	49.4	1.07	.321	ns
SCZ_LoF	10	7.4	1.35	.211	ns
ASD_syn	94	85.7	1.1	.197	ns
ASD_mis	254	192.1	1.32	1.16 × 10^–5^	1.39 × 10^–4^
ASD_LoF	62	28.8	2.16	5.21 × 10^–8^	6.25 × 10^–7^
ID_syn	3	4.1	0.727	.78	ns
ID_mis	16	9.3	1.73	.03	ns
ID_LoF	5	1.4	3.61	.01	ns
control_syn	51	44	1.16	.17	ns
control_mis	109	98.8	1.1	.16	ns
control_LoF	20	14.8	1.35	.11	ns

*Note*: Statistical enrichment for de novo variants in schizophrenia (SCZ), autism spectrum disorder (ASD), and intellectual disability (ID) and differentially spliced genes in *ZNF804A*-depleted cells. De novo variants were collated from Fromer et al^54^ and Genovese et al.^42^ Tests for enrichment were conducted using denovolyzeR.^43^ Initial *P* values were corrected (*P* corr.) for multiple testing (Bonferroni). Only corrected *P* values < .05 were considered statistically significant. ns, not significant.

In conclusion, we present convergent evidence that ZNF804A is involved in the regulation of gene expression and alternative splicing in SH-SY5Y cells. Our data suggest that ZNF804A regulates the expression of genes essential for nervous system development and synaptic contact. These data provide novel insights into the function of ZNF804A and suggest that ZNF804A may interact with splicing factors such as the RBFOX family to regulate pre-mRNA splicing in the brain. Genetic variation in components of this pathway could mediate increased risk of schizophrenia and ASD through concerted changes in alternative splicing and gene expression of important neurodevelopmental genes.

## Supplementary Material

Supplementary data are available at *Schizophrenia Bulletin* online.

sby183_suppl_Supplementary_Data_Set_1

sby183_suppl_Supplementary_Material

## Funding

This study was funded by a Wellcome Trust project grant (WT088866) and the Medical Research Council (Grant No. MR/L010305/1). Chapman and Forrest were funded by Medical Research Council PhD studentships.
