# Supplementary material for: Convergent Evidence That ZNF804A Is a Regulator of Pre-messenger RNA Processing and Gene Expression
Source: Schizophr Bull. 2018 Dec 29;45(6):1267–78. doi: 10.1093/schbul/sby183 (PMC6811834; doi:10.1093/schbul/sby183)
Supplement: sby183_suppl_Supplementary_Material [file sby183_suppl_supplementary_material.doc]

**Supplementary Material**

Chapman *et al*., (2018). Convergent evidence that ZNF804A is a regulator of pre-mRNA processing and gene expression

**Supplementary Methods**

*Molecular Biology*

Plasmids corresponding to each putative interactor were purified and sequenced using standard techniques. Full length myc-tagged ZNF804A and EYFP-tagged Gpatch8 were generated by RT-PCR and sequence verified. Epitope tagged expression constructs were transfected and imaged as described previously ^1^. The 3077 rabbit polyclonal anti-ZNF804A antibody was raised against amino acids 234 of 421 of human ZNF804A fused to thioredoxin as described previously ^2^. Antibodies were immunoaffinity purified against the cognate antigen coupled to Sulfolink (Pierce). A commercial anti-ZNF804A antibody, D-14 (sc-241170), was purchased from Santa Cruz Biotechnology Inc., the anti-myc monoclonal 9E10, was obtained from the Developmental Studies Hybridoma Bank, the anti-ubiquitin antibody FK2 was supplied by Biomol and the SC-35 anti-nuclear speckle antibody was obtained from Abcam. Alexa Fluor-conjugated secondary antibodies were purchased from Invitrogen.

## *Quantitative and reverse transcriptase PCR (qPCR and RT-PCR)*

2.5µg RNA was treated with DNase (Ambicon) and 300ng RNA was reverse transcribed using a ProtoScript M-MuLV First Strand cDNA synthesis kit (New England Biolabs). 2µl of complementary DNA (cDNA) was used for RT-PCR and qPCR as described previously using primers listed in supplementary table S1 ^3^. For analysis of alternative splicing, Primer Express 3.0 (Applied Biosystems) was used to design qPCR primer pairs within the spliced exon and a flanking 3’ constitutive exon. To negate any effects of differential gene expression when calculating the amount of relative exon usage, qPCR primers were also designed in another region of the gene that was not alternatively spliced. Spliced exon levels were normalised to amount of transcript present within the sample. The data is presented as a percentage relative to ratio in the siGAP sample.

**Supplementary Figure Legends**

**Supplementary Figure S1 Initial characterization of ZNF804A and GPATCH8**

Detection of ZNF804A after proteasome inhibition (**A**). myc-tagged ZNF804A was transfected into HEK-293T cells and incubated with the proteasome inhibitor lactacystin (LC). 18h after treatment, protein lysates were prepared for western blotting. Two-channel western blot probed with a custom anti-ZNF804A (3077) antibody raised against amino acids 234-421 of human ZNF804A and the anti-ubiquitin (FK2) antibody which served as a control for successful proteasome treatment. ZNF804A is a nuclear protein (**B**). COS-7 cells were transfected with myc-ZNF804A expression vector and treated with LC (10μM) were labelled with anti-myc antibody (9E10) and Hoechst dye (1μg/ml) to stain for the nucleus. Note the that myc-ZNF804A is detected predominantly in the nucleus. Scale bar, 10µm. Gene structure of the *ZNF804A* paralogue, *Gpatch8* (**C**). The illustration depicts the gene structure (not drawn to scale) of *Gpatch8* showing the location of the poison exons (red) that interrupt the G_patch domain (green). The sequences of exons 3 and 5 are shown. Interesting, exon 3 of *Gpatch8* is also an RBFOX target ^4^. Asterisks identify in-frame stop codons while donor and acceptor splice sites are shown in bold case. Poison exons are also conserved in the human *GPATCH8*. Gene ontology of human G_patch domain containing proteins (**D**). The graph depicts the ontological classifiers arranged in three domains, Molecular Function, red; Biological Process, green; Cell Component, orange. G_patch domain containing proteins are associated with nucleic acid binding and, mRNA processing and the splicesome. Gpatch8 is associated with nuclear speckles (**E**). COS-7 cells transfected with Gpatch8-EYFP were labelled with the anti-SC-35 antibody (nuclear speckles) and Hoechst dye (1μg/ml) to stain for the nucleus. The merged image shows that SC-35 is localized around intensely staining Gpatch8-EYFP punctae in the nucleus. Scale bar, 10µm.

**Supplementary Figure S2 ZNF804A knockdown in SH-SY5Y cells**

qPCR was used to validate siRNA-mediated knockdown of *ZNF804A* (**A**) and *GAPDH* (**B**) in SH-SY5Y cells. Two independent siRNAs (exon 2, siZNFA; exon 3 siZNFB) were used to knockdown *ZNF804A*. Results of four independent experiments are shown. For the siZNF-treated samples, raw Ct values were normalised to *GAPDH* levels in the mock-treated sample. For siGAP-treated samples, raw Ct values were normalised to beta-actin (*ACTB*) levels in the mock-treated sample.

**Supplementary References**

**1.** Forrest M, Chapman RM, Doyle AM, Tinsley CL, Waite A, Blake DJ. Functional analysis of TCF4 missense mutations that cause Pitt-Hopkins syndrome. *Hum Mutat* Dec 2012;33(12):1676-1686.

**2.** Esapa CT, Waite A, Locke M, et al. SGCE missense mutations that cause myoclonus-dystonia syndrome impair epsilon-sarcoglycan trafficking to the plasma membrane: modulation by ubiquitination and torsinA. *Hum Mol Genet* Feb 1 2007;16(3):327-342.

**3.** Forrest MP, Hill MJ, Kavanagh DH, Tansey KE, Waite AJ, Blake DJ. The Psychiatric Risk Gene Transcription Factor 4 (TCF4) Regulates Neurodevelopmental Pathways Associated With Schizophrenia, Autism, and Intellectual Disability. *Schizophr Bull* Dec 8 2017(in press).

**4.** Weyn-Vanhentenryck SM, Mele A, Yan Q, et al. HITS-CLIP and integrative modeling define the Rbfox splicing-regulatory network linked to brain development and autism. *Cell Rep* Mar 27 2014;6(6):1139-1152.

**5.** Zhang C, Zhang Z, Castle J, Sun S, Johnson J, Krainer AR, Zhang MQ. Defining the regulatory network of the tissue-specific splicing factors Fox-1 and Fox-2. *Genes Dev* Sep 15 2008;22(18):2550-2563.

**6.** Yeo GW, Coufal NG, Liang TY, Peng GE, Fu XD, Gage FH. An RNA code for the FOX2 splicing regulator revealed by mapping RNA-protein interactions in stem cells. *Nat Struct Mol Biol* Feb 2009;16(2):130-137.

**7.** Fogel BL, Wexler E, Wahnich A, et al. RBFOX1 regulates both splicing and transcriptional networks in human neuronal development. *Hum Mol Genet* Oct 1 2012;21(19):4171-4186.

**8.** Voineagu I, Wang X, Johnston P, et al. Transcriptomic analysis of autistic brain reveals convergent molecular pathology. *Nature* May 25 2011;474(7351):380-384.

**9.** Fromer M, Pocklington AJ, Kavanagh DH, et al. De novo mutations in schizophrenia implicate synaptic networks. *Nature* Feb 13 2014;506(7487):179-184.

**10.** Genovese G, Fromer M, Stahl EA, et al. Increased burden of ultra-rare protein-altering variants among 4,877 individuals with schizophrenia. *Nat Neurosci* Nov 2016;19(11):1433-1441.

**11.** Ware JS, Samocha KE, Homsy J, Daly MJ. Interpreting de novo Variation in Human Disease Using denovolyzeR. *Curr Protoc Hum Genet* Oct 6 2015;87:7 25 21-15.

**Supplementary Table S1** qPCR primers used to validate gene expression changes in *ZNF804A*-depleted cells

| **Gene** | **Forward sequence** | **Reverse sequence** |
| --- | --- | --- |
| *ACTB* | ACGGCCAGGTCATCACCATTG | GGAGTTGAAGGTAGTTTCGTGGATG |
| *NPY* | GCGCTGCGACACTACATCAA | GGGCTGGATCGTTTTCCATA |
| *CCL2* | GAAGAATCACCAGCAGCAAGTGT | GCTTGTCCAGGTGGTCCATG |
| *SPARC* | TACATCGGGCCTTGCAAATAC | GGGTGACCAGGACGTTCTTG |
| *TMEFF2* | CACAAGGAAATGCCCCAGAA | GATTAACCTCGTGGACGCTCTT |
| *EGR1* | CTTCGCCTGCGACATCTGT | TTTGTCTGCTTTCTTGTCCTTCTG |
| *PDK1* | AGCCATCATTGCACGTGTCTT | CCTTGACCATGCCACTGTACTC |
| *FSTL4* | GTATGCGCTGCTACCAAGATTG | GCAGCTTGGCACAGAAATGAT |
| *EFNB2* | CTGCTGCTGCCTCTGAAACA | CCTACTGGCCTCTTCGATCTCA |
| *ENAH* | GCAGCAAGTCACCTGTTATCT | CTGGACTCCATTGGCACTG |
| *ENAH 11a* | TGTTATCTCCAGACGGGATTC | TCAGCCTGTCATAGTCAAGTCCTT |
| *ENAH const.* | CAGAGTGGTGGGCAGGAAGA | CCCTTTAGGAATGGCACAGTTT |
| *PTPRR 130846* | TTCTCAAGCTCTCATTTAACGT | CTGTAAAGAATCATCAAACAC |
| *PTPRR 002849* | TGGAATTACAGAAGTCTCTCC | GTCTTGTCTTAAGGAAAGCT |

Note. By convention, primer sequences are written 5’ to 3’.

**Supplementary Table S2** qPCR validation of differential gene expression in *ZNF804A*-depleted cells

|  | **exon array** | | **qPCR** | |
| --- | --- | --- | --- | --- |
| **Gene** | **FC** | ***P* value** | **FC** | ***P* value** |
| *SPARC* | 2.29 | 1.25 x 10^-4^ | 2.32 | >0.001 |
| *FSTL4* | -2.06 | 8.32 x 10^-3^ | -2.70 | 6.00 x 10^-3^ |
| *NPY* | 2.05 | 8.14 x 10^-6^ | 3.31 | 1.00 x 10^-3^ |
| *PDK1* | -2.04 | 5.28 x 10^-3^ | -2.13 | >0.001 |
| *EGR1* | 2.65 | 4.49 x 10^-4^ | 3.68 | 2.00 x 10^-3^ |
| *TMEFF2* | 2.37 | 6.80 x 10^-4^ | 2.80 | >0.001 |
| *CCL2* | 3.64 | 4.96 x 10^-3^ | 6.29 | 1.20 x 10^-2^ |
| *EFNB2* | -2.14 | 3.87 x 10^-6^ | -1.90 | 3.00 x 10^-3^ |

Note: qPCR was used to validate differential gene expression in *ZNF804A*-depleted cells.

Statistical analysis was carried out on the ΔCt values using one-way ANOVA and Tukey post-hoc tests. The fold change was calculated by dividing the siGAP-treated ∆∆Ct value by the pooled siZNF-treated ∆∆Ct value. When this number was less than one, indicating a negative fold change, the reciprocal fold change is listed. The summary statistics from the exon array are listed for comparison and are high correlated with the qPCR values (r = 0.98). * = *P* < 0.05 ** = *P* < 0.001 compared to both siGAP-treated and mock samples. FC = fold change.

**Supplementary Table S3** Experimental validation of differential alternative splicing events in *ZNF804A*-depleted cells

| **Gene (exon)** | **Exon** | **Event** | **RBFOX target** | **Validation** |
| --- | --- | --- | --- | --- |
| *ENAH* (11a) | cassette | exclusion | 5-7 | qPCR, RT-PCR |
| *SIPA1L1* (13a) | cassette | exclusion | 6, 8 | qPCR, RT-PCR |
| *PKM2* (9a) | cassette | exclusion | 6, 7 | qPCR, RT-PCR (siZNFB only) |
| *PTPRR* | alt. 5’-end | inclusion | not reported | qPCR |
| *ATP11C* (29a) | cassette | exclusion | 4-6 | RT-PCR |
| *STXBP1* (18a) | cassette | exclusion | 4, 5, 8 | RT-PCR |
| *SEC14L1* | alt. 5’-end | inclusion | not reported | RT-PCR |
| *G3BP2* (7a) | cassette | exclusion | 4, 8 | RT-PCR  (siZNFB only) |

Note: qPCR and RT-PCR were used to validate differential splicing events and alternative transcriptional start site usage *ZNF804A*-depleted cells. For qPCR, statistical analysis was performed as described above (supplementary table S2).

**Supplementary Table S4** Gene set enrichment for DEX genes in *ZNF804A*-depleted cells and neuropsychiatric disease risk genes.

| **Gene set** | **observed** | **expected** | **enrichment** | ***P* value** | ***P* corr.** |
| --- | --- | --- | --- | --- | --- |
| **SCZ_syn** | 5 | 9.2 | 0.546 | 0.95 | ns |
| **SCZ_mis** | 30 | 20.4 | 1.47 | 0.03 | ns |
| **SCZ_LoF** | 3 | 2.8 | 1.08 | 0.53 | ns |
| **ASD_syn** | 41 | 35.7 | 1.15 | 0.21 | ns |
| **ASD_mis** | 98 | 79.4 | 1.23 | 0.02 | ns |
| **ASD_LoF** | 25 | 10.8 | 2.31 | 1.60 x 10^-4^ | 1.92 x 10^-3^ |
| **ID_syn** | 3 | 1.7 | 1.75 | 0.25 | ns |
| **ID_mis** | 9 | 3.8 | 2.35 | 0.02 | ns |
| **ID_LoF** | 2 | 0.5 | 3.83 | 0.10 | ns |
| **control_syn** | 14 | 18.3 | 0.763 | 0.87 | ns |
| **control_mis** | 33 | 40.8 | 0.808 | 0.91 | ns |
| **control_LoF** | 10 | 5.6 | 1.79 | 0.06 | ns |

*Note*: *De novo* variants in schizophrenia (SCZ), autism spectrum disorder (ASD) and intellectual disability (ID) and controls were collated from Fromer *et al*. ^9^ and Genovese *et al*. ^10^. Tests for enrichment were conducted using denovolyzeR ^11^. Initial *P* values were corrected (*P* corr.) for multiple testing (Bonferroni). Only corrected *P* values < 0.05 were considered to be statistically significant (ns, not significant).
